# Food Insecurity: A Constant Factor in the Lives of Low-Income Families in Scotland and England

**DOI:** 10.3389/fpubh.2021.588254

**Published:** 2021-05-19

**Authors:** Jackie Shinwell, Margaret Anne Defeyter

**Affiliations:** Healthy Living Laboratory, Northumbria University, Newcastle upon Tyne, United Kingdom

**Keywords:** holiday hunger, food insecurity, coping strategies, holiday provision, food poverty

## Abstract

During the school summer holidays, pressures on the already tight budgets of low-income families are compounded, particularly when the safety net of free school meals is removed. The main aim of the current study was to investigate how low-income parents and carers feed their families during term time when children receive free school meals and if, and how, strategies differ during the school summer holidays. A secondary aim was to investigate the role of holiday activity and food programmes in supporting parents and carers to feed their children during the school summer holidays. We used purposive sampling to recruit a total of 21 parents (*N* = 20 Female, *N* = 1 Male) whose children attended free summer holiday clubs in Scotland and England during summer 2017. Participants were asked about their food and shopping habits during the school term and if, and how they differed during the school summer holidays when free school meals were not available. The findings suggest that food insecurity is a constant factor in the lives of low-income parents in England and Scotland, and that the stages of food insecurity and the strategies employed to mitigate its effects appear to be cyclical, aligning with the Food and Agriculture Organisation's (FAO) food insecurity continuum and the school academic year. During term time, parents and carers worried about food, suggesting they were experiencing mild food insecurity, despite their children being in receipt of free school meals. As the school holidays approached, moderate food insecurity was experienced as parents reported that they began “provisioning,” storing food and reducing household expenditure. During the summer holidays, food did not last, and parental food acquisition habits became more intense. Parents downgraded food brands and bought reduced price items of food. Ultimately, parents self-sacrificed their own nutritional intake by only buying food their children would eat and parents often skipped meals or only ate their children's leftovers. However, children's attendance at holiday club helped make the food at home last longer and once school resumed, parents returned to their less intense, but constantly coping approach to food shopping. The findings of this study suggest that food insecurity is a constant factor in the lives of low-income families who simply do not have enough household income to prevent them from experiencing food insecurity, even when initiatives such as free school meals and access to holiday club provision with food and activities are in place.

## Introduction

For many low-income families in the UK, the school summer holiday period is a time of dread. Parental stress levels increase as parents and carers face a number of challenges including the cost of entertaining their children and the cost of childcare which increases during the summer break (1–4). However, one of the biggest challenges low income families face during the summer break is the added expense of feeding their children, and this pressure is more keenly felt amongst parents and carers whose children normally receive free school meals during term time (3, 5–9).

Up to 3 million children in the UK are thought to be at risk of “holiday hunger” during the school summer break (5, 7, 10–19). Holiday hunger has been defined as: “a situation that occurs when economically disadvantaged households with school-aged children experience food insecurity during the school holidays” (20). Parents and carers report that they adopt a range of strategies to ensure their children are able to eat during the summer break. Strategies include compromising their own nutritional intake, reducing portion sizes, buying cheaper food items to bulk out meals, eating children's leftovers and, in anticipation of the pressures they will face to ensure their children eat, stockpiling food before the holidays begin (5, 6, 8, 13, 21, 22).

At the time of data collection, a number of local authorities, voluntary sector organisations and charities had either established their own, or provided funding to support a variety of other organisations to set up free holiday club programmes in response to concerns that many children may be at risk of experiencing holiday hunger across the school holidays. The objectives of the clubs were to enable children from low income families to access food and enriching activities during the summer (23, 24). Whilst prior research has demonstrated that holiday club attendance provides a number of benefits including enabling children to access food, and participate in enriching activities, it also reduces household expenditure and attenuates household food insecurity (13). The aim of the current study was to investigate the strategies used by low-income parents and carers to feed their children across the academic year and if, and how, strategies differ across term time and school holidays. A secondary aim was to investigate the role of holiday club provision in supporting parents and carers to feed their children during the school summer break.

## Materials and Methods

### Approach

Semi-structured interviews were used to collect data in this study. This approach is considered inclusive and allows participants to share their views regardless of age or educational ability and enables participants to talk freely and openly about sensitive topics such as food insecurity (25). Interviews were carried out face to face on holiday club premises.

### Participants

Twenty-one parents and carers (*N* = 20 female, *N* = 1 male) were recruited to this study using non-probability purposive sampling. Participants were parents and carers of children who attended holiday clubs that were part funded by the Meals & More charity, which awards grants to community organisations and schools to provide free holiday clubs with food and activities during school holiday periods for children from low-income families. All participants attended holiday clubs that were located in Scotland and England where similar free school meal policies and broadly similar models of holiday club provision, provided children with a free meal and access to a range of physical and cultural activities. Demographic characteristics of participants are presented in Table 1 and details of the holiday clubs (*n* = 10) that participated in this study are presented in Table 2.

**Table 1:** Demographic characteristics of participants.

**Demographic**	**Response option**	**Number of parents/carers**
Sex	Male	1
	Female	20
Age range	18–25	1
	26–35	14
	>36	6
Marital status	Married/living with a partner	12
	In a relationship but living apart	1
	Single	8
Income	< £15,000	15
	£15,001–£24,999	4
	£25,000–£34,999	0
	>£35,000	1
	Declined to say	1
Employment status	Unemployed	13
	Employed—Full time/self employed	4
	Employed part time/0 h	3
	Retired	1

**Table 2:** Holiday club characteristics.

**Club**	**Setting**	**Operational hours of clubs**	**Country/Region**	**Index of multiple deprivation rank**
1	Community	11.00 a.m. −1.00 p.m. 5 days a week × 6 weeks	Scotland	SC1119
2	School	10.00 a.m. −3.00 p.m. 5 days a week	Scotland	SC1536
3	Community	11.45 a.m. −1.30 p.m. 5 days a week × 1 week and 1 day a week × 5 weeks	North East England	2,803
4	Community	10.00 a.m. −4.00 p.m. 5 days a week × 6 weeks	North East England	6,435
5	Children's Centre	10.30 a.m. −1.30 p.m. 2 days a week × 4 weeks	East Midlands	18,236
6	Church	12 noon−1.30 p.m. 2 days a week × 6 weeks	East of England	25,830
7	Church	11.00 a.m. −1.00 p.m. 1 day a week × 6 weeks	East of England	2,332
8	Community	11.30 a.m. −1.25 p.m. 4 days a week × 6 weeks	London	4,275
9	Community	11.00 a.m. −1.00 p.m. 1 day a week × 6 weeks	North East England	376
10	Church	11.15 a.m. −1.05 p.m. 2 days a week × 6 weeks	North East England	5,860

### Materials

A semi-structured schedule of questions was developed to enable parents to talk freely about how they shopped for food and fed their children during the school term and the school summer holiday. A copy of the schedule of questions is presented in Table 3.

**Table 3:** Schedule of questions.

What is your normal shopping routine during term time?
Does this change during the holidays? If so, what changes do you make?
Do you buy different food or shop in different places during the holidays compared to term time?
Do you change what you and your family eat during the holidays compared to term time?
If you do not have enough money for food what do you do?
Have you ever borrowed money to buy food?
Have you your children to friends or relatives houses for food during the term/during the holidays?
Have you ever skipped meals so that your children can eat?
Have you ever visited a food bank?

### Procedure

Following ethical approval from the Faculty of Health and Life Sciences Ethics Committee at Northumbria University, Newcastle upon Tyne (Ethics reference number 300317), information about the study was distributed to sixteen holiday club leaders whose summer holiday club was part funded by the Meals & More charity. Ten club leaders expressed an interest in taking part and were sent a research information pack, a letter of invitation, a research information leaflet and an opt-in consent form. Following the receipt of consent, holiday club leaders distributed letters of invitation, research information sheets and opt in consent forms to parents/carers whose children attended their holiday club. Only parents who had provided written consent were interviewed. All interviews were conducted face to face by a researcher from Northumbria University and all participants were advised that they could withdraw their consent to participate at any time and that they did not have to answer any questions they were uncomfortable with. Participants were advised that all data would remain confidential and all data would be anonymised. Prior to recording, permission was gained from all participants to record their voice. All interviews followed a semi-structured format which enabled parents and carers to talk freely about how their experiences of shopping for food and feeding their children during the summer holidays and whether it differed compared to term time.

### Analysis

Data transcripts were the main unit of analysis. Each interview was listened to in its entirety before being orthographically transcribed. All data were uploaded into Nvivo 12 for ease of access and organisation. Transcripts were read several times to gain a thorough overview of the data. Data were coded and analysed in accordance with guidelines for grounded theory developed by Strauss and Corbin (26). Analysis of the data commenced upon initial data collection with open coding. Following this stage, a bank of codes and memos were compiled in Nivo 12, and subsequently codes were organised and merged during a deductive process to reduce redundancy and aid organisation. The final phase of analysis was axial coding, whereby codes were subjected to a comparative analysis and relationships were identified between codes, then further reconstructed into a smaller number of larger tentative categories and themes.

## Findings

**Figure 1:**
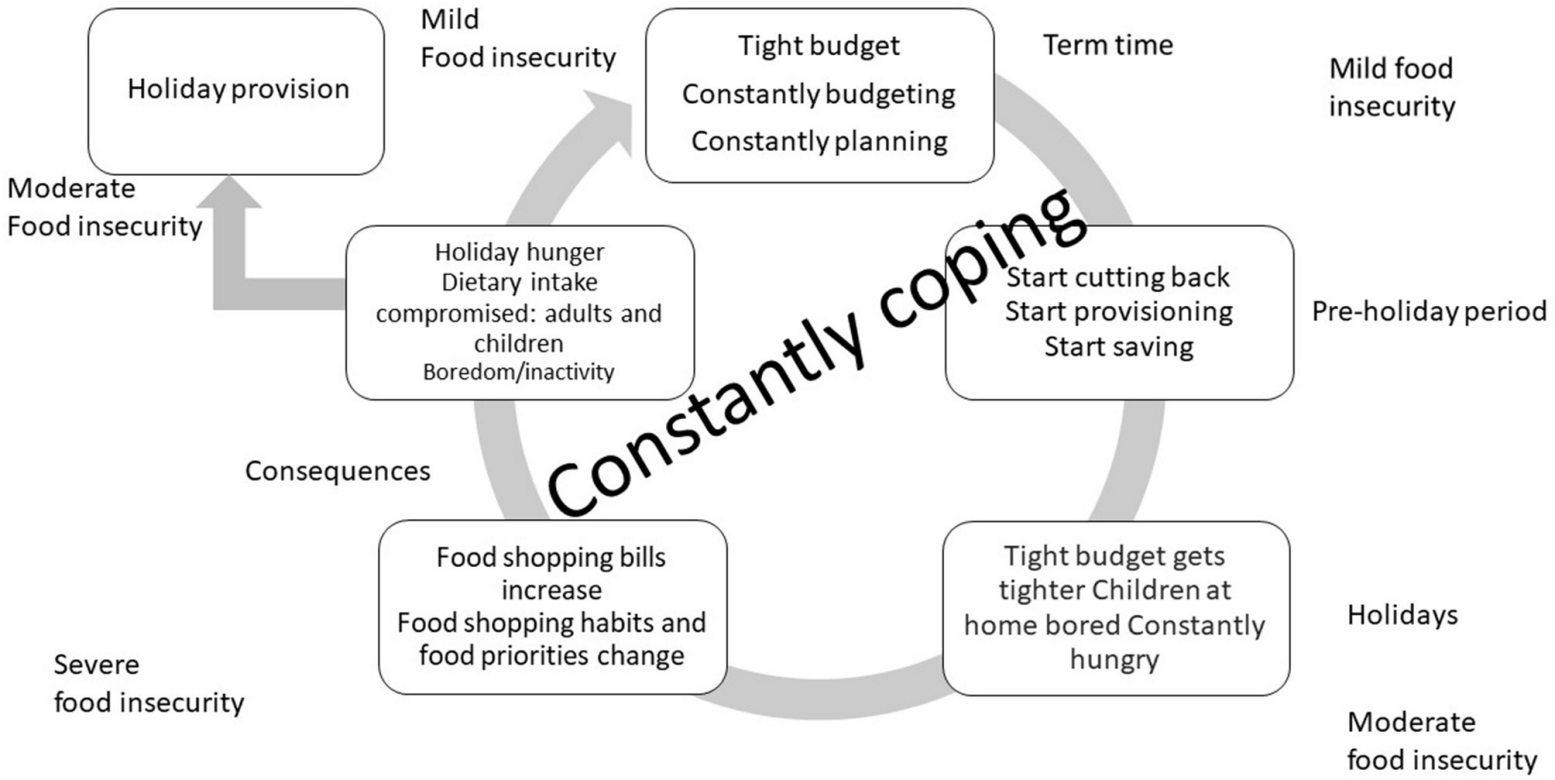
A model of how parents and carers of child holiday club attendees food insecurity experiences change during the school academic year and school summer holiday period, based on the FAO food insecurity continuum (27).

Participants provided a unique insight into how they shop for food and feed themselves and their children throughout the academic year. Three broad themes were identified which related to distinct periods of time: term time, 1 month before the school summer holidays (pre-school summer holiday period), and during the 6- or 7-week summer break. The three broad themes and associated sub-themes are presented in Figure 1 below which is based on the Food and Agriculture Organisation's (FAO) food insecurity continuum. A detailed analysis of each of the main themes and associated sub-themes, together with example quotes from participants, are presented below. Quotes have been anonymised and are represented by the letters PC (parent/carer) and a number to identify the source of each quote.

### Term Time

Parents and carers reported that during the school term they constantly juggle their finances and often worried about their ability to feed their children. Although the provision of free school meals during term time helped parents cope, parents still employed a number of strategies to attenuate food insecurity. Sub-themes identified included having limited financial resources and the need to constantly juggle and manage their money, use of foodbanks, food shopping habits during term time, and meal planning and free school meals.

#### Limited Financial Resources and Constantly Budgeting

For many parents and carers, managing budgets and juggling competing demands for their limited resources was a constant challenge, with one parent/carer summing up the struggles that many seemed to face by saying:

“From the moment you wake up in the morning just thinking about the whole day is just money, money, money, life is a struggle, it's really hard.” (PC4)

When faced with competing demands for limited resources, some parents reported that they endeavoured to live within their means, with outgoings paid by direct debit as soon as they received their income. Ensuring that families kept a roof over their head was one of the most important priorities. However, when faced with demands for other bills, parents needed to juggle their finances. Ensuring children were fed was a priority, as explained by one parent who said:

“*I think yeah I have learnt to budget more and erm realise that if I haven't got the money for a bill I can't pay it or if the kids need more, if we need more food and cannot last a couple of days or whatever, I would rather buy food than pay a bill that week or whatever, so it depends on the situation at the time, if I think we can last another couple of days on food I won't buy any and I will pay something, do you know, so it depends on the situation at the time but yeah I always make sure there is food in the house definitely.” (PC10)*

In a similar vein, another parent said that feeding and clothing her children was a priority, but it had sometimes been necessary to feed her children cold food out of a tin because of the cost of using energy to heat food:

“*you need the gas and electricity like to cook the food and stuff, like I mean sometimes the kids have had to have microwave meals or like beans and sausages out of the tin and stuff for their dinner and teas because we haven't got enough in that week and stuff.” (PC18)*.

Another parent recalled how a relationship breakdown and problems with receiving benefits had left her with a reduced income of just £30 per week, but feeding her children was a priority and she would rather sit in the dark to ensure her children were fed:

“*I would rather not have a light on than have a hungry child so yeah, even if it was just chicken nuggets and chips, as long as they had something that they would eat, the bills was not even in my mind especially like with £30, because if you think, £30 is nothing. You pay gas, electric and you've got £10 (left) from that.” (PC19)*.

Nevertheless, for some parents, money simply did not last. One parent recalled how despite her best endeavours to plan and budget, money and food would both be in short supply before her next benefit payment was received, reflecting the cyclical nature of food insecurity even on a week-to-week basis for some low-income families:

“*Monday always is a really hard day for me because I get my money on a Tuesday, so it is the last day and I've gone, I normally run out of money by a Friday so the whole weekends are normally like, it's juggling you know, looking at what I've got and you've got to pre (plan) like what meals have you got, what you can put together? So today like I have probably only got one more meal in my freezer.” (PC15)*

#### Use of Foodbanks

A number of parents and carers reported that they had used foodbanks in the past because they had run out of money. They reported that their disposable income was depleted because of high levels of debt, difficulty in paying bills, and the failure of the welfare state to provide adequate support:

“*The reason why I went to the foodbank (was) because my tax credits and obviously at the financial year there was no money, and they took £120.00 off me a week and I had to go to the foodbank so that I could feed us.” (PC02)*

One parent reported that she worked 30 h a week, on a minimum wage but did not qualify for any benefit support from the government. High levels of debt meant that she had needed to use a foodbank to feed her children:

“*Obviously I work full time and I was in a considerable amount of debt last year and it got to the point where it became really overwhelming and it was either do that or we didn't eat, and I think people underestimate that you could be working and earning as much as you can but if you are on hourly pay and on minimum wage or living wage in this case as I am, you can still struggle because there is no help for someone who works 30 hours a week in terms of benefits, I don't get a lot of support when it comes to financial things and I ended up with lot of people saying you owe us money and we want this and nobody considered that I had me and a family to feed.” (PC12)*

Visiting a foodbank was considered a strategy of last resort and was only used when there were no other options available. Nevertheless, one parent who had been referred to a foodbank by a domestic violence support organisation decided not to go because she thought there were people who needed it more than she did and said she would rather take out a crisis loan than take food bank resources:

“*(Name of organisation) first referred me over to a food bank but I didn't want to take advantage of that because there are more people out there that need it more than me so I didn't, I wouldn't go to somewhere like that because there is people that really need it and are homeless and have nowt (nothing) so to me, I wouldn't use somewhere like that, I'd rather get a crisis loan and pay it back so that I'm not taking.” (PC18)*

#### Food Shopping During Term Time

When asked about their food shopping habits during the school term, parents and carers indicated that they used a range of different strategies, many of which were ultimately designed to save money. Shopping while children were in school or left with relatives reduced “pester power” and made shopping easier, otherwise as one parent explained, if her children were with her when she shopped, the food bill would increase:

“*when you take them with you, they stick everything, things in the trolley as well which costs you more money.” (PC01)*

Some parents reported that busy morning and family schedules meant that pester power was reduced during term time. One parent for example explained that:

“*because of the length of stuff, they've got to do in one day and the length of stuff that we have to go through just to get them out of the door in the morning, we just don't have that time oh let's go to that sweet shop or let's go and buy.” (PC04)*

Similarly, another parent who worked full time said that she chose to do her shopping online during term time, not necessarily because of time pressures, but because it reduced pester power and meant that she did not have to “drag” bored, hungry children around the shops, commenting that:

“*sometimes I will take them shopping but I just have to make sure that they are in the right frame of mind and they're not hungry but generally I do an online shop.” (PC13)*

When parents and carers did go shopping, many reported that they visited multiple grocery stores, particularly discount supermarkets. Finding bargains was an important aspect of parents' and carers' food shopping habits. One parent for example, explained that she received information about discounted prices from friends and family and as a result, would visit stores she would not normally shop in to purchase a bargain:

“*Everybody likes a good buy, and I am not going to say that I don't like my bargains, I love my bargains. I will do shop comparisons, I'll go to one shop if one shop has got it cheaper, I will definitely buy it in that shop, so if friends or family have said there is such and such on offer, if I don't normally shop at (supermarket) I will go in to (supermarket) just to get you know, if it's a good buy and stuff.” (PC05)*

However, parents were careful not to buy products just because they were on offer:

“*I am a bit of a sucker for the 3 for 2, but I do try to keep to store own brands just to keep the cost down where I can, but yes, if there is something on offer, I am definitely drawn to that, but it has to be something that I use. I wouldn't just get it for the sake of it and it is not just something that is going to go to waste. But definitely things like cheese and like yoghurts that kind of thing.” (PC13)*

One parent also indicated that she timed her shopping trip to take advantage of the “oopsie” offers, where the price of fresh food approaching its best before/use by date was reduced:

“*…I can't stand any waste of food, so what I tend to do is shop on the day for the day. If you go to certain supermarkets you can find out what time they do their reduced, like (name of supermarket) here, they put their reduced stuff out at 7 o'clock, between 6 and 7, and it is dirt cheap. I mean you literally get strawberries for 20p. I have gone in there and have literally spent £5.00 and come out with 4 bags full of shopping and as I say, I will go home and put all of that fruit on the table and the kids will graze on it.” (PC15)*

#### Meal Planning and Free School Meals

Reducing and preventing food waste during term time was important for many parents. Some parents said this was achieved by planning meals in advance:

“*I plan a week in advance. I go and get the meat, I go and see what they have got and then I get it and I then I plan, I make a menu of erm what I am going to cook every night and what kind of ingredients that I need to cook to make sure that I have everything.” (PC17)*

Importantly, parents and carers said they only needed to concentrate on providing food for evening meals during the week during the term because children were able to access free school meals:

“*Yeah, that's it, during the week my kids are all young and they all get free meals so I don't pay for their lunch, like every day they're eating, they're getting a cooked hot meal a day from school.” (PC10)*

### Pre-school Summer Holiday Period

Parents and carers were acutely aware of the timing of the school holiday periods and many endeavoured to prepare in advance to ease the anticipated pressures they would face. Two key themes of provisioning and saving money were identified as strategies used by parents and carers to prepare for the school summer break.

#### Provisioning

In anticipation of their children being at home for long periods of time several parents and carers indicated that they start stockpiling and hoarding food in the run up to the school summer holidays. For some, it usually started around 3 or 4 weeks beforehand and included stocking up on dried foods such as rice, pasta, and tinned foods. One parent described the freezer as her “*best friend*” (PC17), thus freezing food also formed part of the tactics used by some parents to prepare for the summer holidays:

“*I start hoarding before the holidays. Anything like dried stuff and tinned stuff all in the cupboards like soup and stuff to get through the day. I actually put bread in my freezer because during the week we don't actually eat a great deal of it during term time, so I keep it in the freezer so a loaf of bread will last about 2 weeks in my house, but I would usually have about 3 or 4 loaves in the freezer by the time summer hits, and that's what I mean by hoarding I try and ferret or hamster it away or whatever it is called.” (PC12)*

Another parent said that they also endeavoured to stockpile some fresh foods before the summer:

“*we don't do it all the way through the year, it becomes, I would say in the middle of June when the summer just starts … we start buying bulks of stuff, potatoes and onions and stuff, bigger stuff which we wouldn't do through the year anyway because we are just so busy, but we just always think ahead in these kinds of months, thinking things are going to get really rough.” (PC04)*

The same parent also commented that they stockpiled other items such as washing powder and bleach because children would be at home showering more and the house would need to be cleaned more frequently. However, they indicated that they downgraded the brands of these household products bought during the summer, or bulk bought some of these items to enable money to be saved for unanticipated expenses:

“*we actually bought big bulky stuff like that and it's lasted right through yep. It just shows just to save that extra £20.00 and what you actually have to do for your family to save that extra £20.00 because that £20.00 you might need that tomorrow.” (PC04)*

#### Saving Money

In addition to stockpiling food in advance of the summer holidays, some parents and carers indicated that they also tried to save money in the run up to the summer break in an attempt to meet the costs of the school holidays:

“*I tend to try and save so that I have a bit of money put by for holidays because otherwise you do, you find that it's so thin.” (PC16)*

“*normally about three or four weeks before I think oh, I will say oh I'll keep, I'll stay back, I won't get this, I don't really need that, try and keep a few things back and then I'll, then you (know) when the kids are off then we can do this and do that because we have a few quid spare now.” (PC07)*

Some parents indicated that they put “spare” money out of physical reach by for example, putting it into money boxes:

“*what I do is you (know) them money boxes that you break, I save up in them in between each school holidays and then I break it in the school holidays so that I have extra money for them in the holidays, and what is left over we'll go on a day out or something.” (PC18)*.

Another parent explained that she gave any spare money to her mother to look after so it was available to spend during the summer holidays when she most needed it:

“*I normally give it to my mum so then obviously, if we want to do things then I have got it.” (PC16)*

### Summer Holidays

The school summer holidays presented significant financial challenges for parents and carers. Demands on their limited resources increased but there was no commensurate increase in income to meet these additional needs. Seven main themes of: tighter budgets and increasing food shopping bills; changing food shopping habits during the summer holidays; changes to the nutritional value of meals served during the summer and reducing food waste; parental self-sacrifice; support networks; the cost of entertaining children during the summer holidays and free food and activities were identified which reflected the challenges and coping strategies used by parents and carers during the summer holiday period.

#### Tighter Budgets and Increased Food Shopping Bills

A number of parents and carers indicated that their already tight financial budget got even tighter during the summer holiday because they did not receive any extra financial support to cover the additional costs encountered during the summer break. The sentiments expressed by one parent were echoed in interviews with other parents:

“*we don't get any extra money and you just about get by during the week on what we've got and then you know also during the holidays it's, and the kids want to do stuff, and can we go here, can we go there, and I feel like I am constantly saying no because I just can't I don't have the money.” (PC15)*

One of the main challenges parents and carers faced was finding the money to meet the cost of providing the lunchtime meal during the week for each of their children, as one parent explained:

“*Obviously you don't realise when they're at school and when they don't, when they are not at school it's that transition and it's breakfast, dinner and tea whereas it's just breakfast and tea at home when they are at school.” (PC19)*

As a result, food shopping bills increased:

“*it is usually easier to plan food shopping when they're at school, so the food bill goes up and things get really tight.” (PC12)*

There was some variation in how much the cost of buying extra food added to weekly shopping bills during the summer holidays. Some parents/carers said their shopping bill doubled, costing up to an extra £50 to £60 a week. One parent suggested the family food shopping increased by up to £100 per week during the summer and another parent, who shopped monthly said that the shopping bill increased by nearly £300 per month compared to a normal monthly spend of £150:

“*Normally I get like fridge stuff during the week and that and I usually do my big shop on a monthly basis but since obviously when they come out of school instead of my shopping bill being £150 it usually goes up to about £430.” (PC16)*

Parents and carers indicated that they were just as careful with their grocery shopping during the summer holidays as they were during the term. One parent explained that the £5 she saved per week because her children did not go to Brownies during the holidays was used to buy things in the summer break:

“*during the holidays it's harder but I will be as savvy as I am during the holidays as I am during the week because we don't just have enough money and any money that we can save during the week as …at school time the kids go to Brownies and Rainbows and that is £5.00 a week that I've got to find, I don't have to find that money during the summer holidays so that is something extra towards our budget during the week and the clubs things.” (PC15)*

#### Changing Food Shopping Habits During the Summer Holidays

To cope with the increased cost of grocery shopping, parents and carers reported that their food shopping habits changed during the summer holidays. The underlying principle behind these changes was to try to ensure that there was food at home and that it lasted. Tactics included down grading food brands, changing the type of foods purchased, and frequency of shopping:

“*I am having to go shopping maybe 3, 3 times a week whereas in term time I go once or maybe sometimes I go at the beginning of the week and then I fill up, I top up on a Thursday when I am off work.” (PC17)*

There were two main reasons for increasing the number of times parents and carers shopped. Some parents indicated that the food they had did not last, so they visited shops to top up on items such as bread and milk more frequently:

“*we would do shopping on a Saturday but during the week we are nipping to [name of supermarket] we're nipping here, we're nipping there it's oh this is finished oh that is finished.” (PC05)*

Other parents and carers said they shopped more often as a way of making food at home last longer, explaining that once food was brought into the home, it disappeared very quickly. Shopping more regularly and buying smaller amounts of food compared to a regular big shop meant that food lasted longer, and money could be used for other things:

“*I'm doing bits at a time because if, I find if I just get loads every, all the good stuff is taken and then you just end up with whatever you've got left do you know what I mean, like I am doing £20 shops every now and again rather than big shops and it is working out better…. it seems that I have more cash there if we need to, if we go to play group or if we are going somewhere like planning trips out and stuff like that.” (PC07)*

Some parents did their food shopping online during the summer holidays. In some instances, parents shopped online because their children did not like to go food shopping. Other parents reported that online shopping reduced pester power, whilst other parents reported that it enabled them to monitor how much they spent and allowed them to easily remove items not considered vital out of the online shopping basket:

“*the luxury things like crisps or whatever that you don't need and keep in what you really need kind of thing.” (PC01)*

Some parents and carers downgraded the food brands and the type of food they bought during the summer:

“*You see I go for the cheap things in the summer because it doesn't last long because it is like family choice or whatever the cheap (name of supermarket) or (name of supermarket).” (PC02)*

One parent explained that she bought the cheapest bread she could. One shop she said, sold bread for 79p a loaf, compared to £1.30 elsewhere, so she bought the 79p loaf. The difference in cost could be used towards the cost of buying another loaf of bread:

“*that's because I know they're going to eat more of it and it's going to run out faster and then I am going to have to by another one, and that 41p that I have saved I can use that again tomorrow. It's the way we have to think, it's the way we all think like. I think the majority of us think like that, they're going to ask for that again so why not just have it, buy it cheaper and then we can buy it again.” (PC04)*

A parent from another club explained that during the summer holidays she bought cheaper sandwich fillings:

“*Normally I would go for things like [name of child] would normally have like a cheese sandwich but it is things like chicken paste or cheap chocolate spread and peanut butter and cheap jam, they are all cheap enough to buy but you can get quite a bit and you do and bags of crisps.” (PC12)*

As well as down grading brands and products, parents bought different types of foods, in particular more snack type foods:

“*a lot more snacks, because if we go out for the day or maybe we have come here for the afternoon, so I am just making sure that I buy a lot more snacks just to keep them going throughout the afternoon.” (PC13)*

This was, as a number of parents and carers explained, because their children were constantly eating when they were at home because they were bored. As parents could not afford to do other activities outside the home, days were less structured, and children stayed up later which often resulted in children eating snack food late at night, as one parent explained:

“*they are constantly, when they're at home even though they are not hungry they say they are hungry because they are bored and they're not doing stuff if, if you're not doing anything because everything that you do costs a lot of money…” (PC18)*

Some parents bought healthier snacks that increased the cost of their shopping, but they still tried to keep costs down:

“*they are eating a lot more snacks because they are hungry more through the day. I am trying get everything healthy trying to do everything as low cost but healthy.” (PC07)*

Although money was tight, parents felt it was important to buy small treats for their children:

“*I notice that we are buying loads of ice pops, that's something our freezer gets filled with definitely in the summertime, lots of juices and lots of cans, lots of water bottles, well sorry not cans, we don't have fizzy juice but lots of little cartons of juice and stuff.” (PC05)*

Paradoxically, although parents had to spend more money to have ice lollies and similar products at home, this meant that parents and carers actually saved money as the cost of buying similar treats from ice cream vans was even more expensive. Having a ready stock meant that children did not miss out:

“*if you haven't got them in the freezer then you are paying £2.00 each for the ice cream man and all this kind of stuff and if your kid is out and somebody else's kid is getting an ice lolly and they are not, so you've got all that expense all of the time and it builds up.” (PC11)*

#### Changes to the Nutritional Value of Meals Served During the Summer Holidays and Reducing Food Waste

When parents were asked about the type of food they served their children at mealtimes during the summer holidays, many parents explained that they generally provided a lighter, picnic type lunch, consisting usually, for example, of sandwiches and crisps. They tended to provide a hot meal for their family's evening meal, as they did during term time. A comment made by one parent reflected comments made by many others that cost was the main reason for this:

“*they [children] will have like sandwiches or beans on toast so stuff that is easy and cheap because you can't afford to feed them all three meals a day because it costs too much.” (PC18)*

Another parent indicated that the nutritional value of the food she fed her children declined during the summer because her budget was so tight:

“*when you've not got a lot of money, like I can feed my kids on a couple of quid [British pounds] (a day) if I buy them chips or fish fingers or reduced like foods yeah, but it's not, none of that is nutritional, got any nutritional value for my kids so you know, it feeds them but it's not really a good meal for them.” (PC15)*.

Another parent indicated that she would try to pad meals out to fill children up:

“*I normally do like, if I do like pasta, I would put sausages in or hot dogs you know like to fill them up more, or with noodles I do hot dogs.” (PC10)*

Preventing food waste and incorporating left over food into other meals was a strategy used by some parents to make food go further:

“*Say that the food, like If you made a big bowl of soup that you know, soup doesn't go off you can keep it in the fridge, you can eat it for two days and it is fine. This only starts happening between these weeks still, we, so she made rice last week right so I said, she was just about to bin it and I (said) don't bin it, you can freeze I, it's just rice you can freeze rice. She freezed it, two days later, took it out of the fridge used it and we used it again and that was their tea.” (PC04)*

Some parents indicated that using leftover food occurred more frequently during the summer:

“*Say if I have made lasagne the night before mbbe [maybe] if there's any left we will do that for dinner or something the next day, maybe one of them will have that for dinner if one of them really wanted it so it's just doing it that way whereas normally, we would have just thrown it.” (PC06)*

#### Parental Self-Sacrifice

It was evident that many parents and carers prioritised the need to feed their children and grandchildren over the need to feed themselves, and multiple instances of parents/carers sacrificing their own needs were reported in interviews. These acts of altruism occurred across several dimensions in relation to coping with the demands of the school summer holidays. Examples included parents and carers reporting that when they shopped for food, they only bought food their children would eat and only cooked food their children would eat and never bought anything that could be perceived as a luxury for themselves, as exemplified in the following quotes from two parents/carers:

“*So, when I go for the shopping, I don't really shop for myself either I just get what the kids want first and then I am second thought, so whatever money is left.” (PC19)*“*Well, it's hard, it is hard. No treats for mum that's what it is, I get no treats in the holidays.” (PC17)*

A grandparent who cared for her grandchildren during the holidays so the children's parents could work described how she did not like to ask for money for food for her grandchildren. Instead, her and her husband, who both had multiple health conditions cut back on all luxuries, including food, during the summer:

“*It is stuff like the little treats that we change and stuff that we will do without, again you might have yourself a little pork chop to go with a bit veg and potatoes and whatever but them sort of things stop you know, you can't do it.” (PC11)*

Similarly, there were several instances where parents reported that they either ate their children's leftover food or simply did not eat some meals during the holidays, sometimes up to as many as three or four times a week because they did not have enough money for food. One parent acknowledged that not eating caused her to feel tired:

“*I do tend to just feed the children and then I might just pick off their plates if I like it cos, so picking for me is, or I will just have a couple of biscuits with a cup of tea or something, so and it's not, I know it does, it sort of tires me out a lot not eating like I realise I have, because I don't eat breakfast or dinner so I just have tea.” (PC19)*

Another parent reported that she sometimes did not feed herself just because she had no money and in rare instances, her children occasionally went without some items of food:

“*if we're starving if we're starving then I will eat but yeah I have gone without when there's been no money, I've just fed them and then they've gone without as well which saves, they never, they never go without their dinner but they might go without their yoghurt or something like that if we are having a bad week and like my kids know and I just say sorry I just haven't got it this week.”(PC17)*.

#### Support Networks

Some parents and carers sought support from family and friends during the summer holidays. Sometimes it was for childcare so that parents could go food shopping. However, some parents said they often visited their own parents' house for a meal. One parent for example said that if she went to her mother's house, both her and her daughter would get a cooked meal which had the added benefit of saving food at home:

“*We go round there sometimes to sleep over because I know then my mum will cook us food and it gets us out of the house you know, and it saves food at my house and I know (name of child) will get a cooked meal.” (PC14)*

Another parent explained that following a traumatic relationship breakdown, her mother had moved in with her temporarily to offer her support over the summer. Her mother prepared and cooked meals for her and her children, and without that support, it was highly likely that she would have not eaten anything:

“*I mean it was from May until August that this was going, so she has been, so if it hadn't, if it wasn't for her and she cooks a lot for me. I think because there is someone to eat with as well, normally I am on my own and I'm like nobody will know if I don't have this meal.” (PC19)*

Elsewhere, a parent explained that she and a friend provided each other with extensive support to cope with the demands of the holidays. The friends often came together to share childcare, but most importantly food to enable their children to eat:

“*I'll say to her, what are you having….and she hasn't got anything, and I have only got what I have got, then we will just feed the kids, that's what we will do, we will eat their leftovers quite often or a sandwich….” (PC15)*

#### Cost of Entertaining Children During the Summer Holidays

Parents and carers suggested that they had to be extremely well-organised to find money for extras during the summer holidays. One parent said she had a strict timetable to meet these demands:

“*money, money is a big issue when they are off as well because they constantly, if they see an ice cream van, they see this and see that, so it is constant from head to toe. You just have to have a plan; it is almost like having a timetable and a budget timetable as well.I would say (we spend) at least an extra £50/ £60 per week which is a lot.” (PC04)*

When money was not available, many children were often confined to the house. One parent said that when she did not have any money, she had the difficult task of telling her children they could not do anything or go anywhere:

“*We would just have to sit in the house and basically get on with the day and there would be nothing else we could do and it is really hard telling your children that we just can't do it, or even turning round to say sorry kids we can't afford this today because we have this on next week, or we have a bill coming in or something but you just have to stand your ground and tell them straight up this is what is happening, but it is horrible doing that because I've done that.” (PC19)*

Another parent indicated that she went into debt and sometimes borrowed money in order to treat her children during the summer:

“*Just because they are always wanting something or that, or to take them somewhere, it costs a fortune to take them somewhere you know, so I don't have that much left-over kind of thing, so I borrow money to take them, say like to the soft play one day or swimming kind of thing, just for a wee treat kind of thing.” (PC01)*

#### Holiday Provision With Free Food and Activities

Due to the tight financial constraints parents and carers faced during the summer holidays, many reported that they actively sought out free activities, such as going to parks and museums and free holiday provision. They also tried to save money by taking packed lunches. However, a days' activities that on the face of it appeared free, often turned out to be expensive. For example, if there was an ice-cream van at a park children would often pester their parents for an ice-cream. Therefore, free holiday clubs helped to reduce the financial strain on low-income households by providing children with at least one free meal and opportunities to participate in a range of activities, as one parent/carer explained:

“*well, I use things like this the holiday clubs, like find somewhere where they do like a, like last week we went to a barbecue that was free like at the church so to make the food stretch a bit more we just go out like and see if there is anywhere that does low cost like this and a free dinner or you know like go to a free barbecue or something like that.” (PC10)*

One parent, who worked part time, indicated that she wished there were more clubs to go to as the club she visited only opened twice a week and she had to work on one of the days. Nonetheless she welcomed the support holiday club provided during the summer:

“*For me as a working parent it definitely helps you know my situation and I wish it was on more often and I wish there were other ones for us to got to but other than that I am happy with you know this.” (PC14)*

## Discussion

The findings of the current study present a unique insight into the year-round strategies used by low-income parents and carers to feed their families. The findings suggest that food insecurity is a constant factor in the lives of many low-income parents and carers in Scotland and England and that the stages of food insecurity and the strategies employed to mitigate its effects appear to be cyclical and align not only with FAO food insecurity continuum, but also with the school academic year. When schools are open, parents and carers reported being worried about food, suggesting they were experiencing mild food insecurity, despite their children being in receipt of free school meals. However, as the school summer holidays approached, parents started cutting back on expenditure so they could stockpile food and save money in anticipation of the forthcoming challenges of the school summer holidays. Once schools were closed, parents were presented with a conundrum. On the one hand, they did not have money to spend on activities outside of the home environment, but this meant that children would be at home bored, resulting in increased demands for food and snacks which cost money. Because parental incomes did not increase during the summer holidays, but they still have to find the funds to meet the cost of buying extra food, parents' and carers' food shopping habits changed in the holidays. They downgraded brands and the type of foods bought and visited families or other support networks to access food. Ultimately however, parents and carers self-sacrificed their own dietary intake in favour of their children's. Parents actively sought free activities, such as holiday provision with food and fun activities, to attenuate the effects of food insecurity.

Prior research has demonstrated that food insecurity is a dynamic, and sometimes cyclical process: people may move in or out or along the FAO food insecurity continuum as income becomes depleted, for example towards the end of the month when money and food stamps are running low. As food insecurity becomes more severe, strategies used to cope become more severe (28–32). Prior research has also shown that the school summer holidays, and other periods of prolonged school closures represent a pinch point when food insecurity increases amongst low-income families who struggle to meet the cost of providing extra food to feed their children. As a result, parents adopt a range of strategies to cope, including stockpiling food and eventually skipping meals (6, 13, 18, 19, 30, 33). However, the findings of the current provide new insights into how low-income families in Scotland and England experience food insecurity and suggest that food insecurity is cyclical and aligns with the academic year. In addition, the findings suggest that dietary intake may begin to be compromised up to 1 month before the school summer holidays commence when parents anticipate the struggle they will face over the summer holidays and start to reduce expenditure and begin to stockpile food.

During term time, free school breakfasts and free school lunch programmes improve children's nutritional intake and may also reduce obesity (34–41). The introduction of Universal Infant Free School Meals, where all primary school children in reception and years 1 and 2 in England and years P1–P3 in Scotland receive a free school lunch time meal saw the greatest take up amongst low income families and saved families an average of £10 per week per child (42). However, the safety net of free school meals is removed during the summer and an estimated 3 million children are thought to be at risk of going hungry (5). In response to these concerns, a number of organisations have established holidays clubs to enable children to access free, healthy food during the summer, and the number of organisations doing so has increased at a rapid rate and clubs are highly likely to be located in areas of high deprivation (23). Moreover, since data were collected for this study, the UK government and the devolved administrations in Scotland and Wales have acknowledged that there is a need for this kind of provision and have made funding available to support the establishment of more clubs (23, 24, 43–45). Attendance at such holiday clubs has a number of benefits including reducing food insecurity, and stresses faced by low-income families during the summer and thus having a positive impact on children's nutritional intake and broader well-being (8, 13, 46). However, the findings of the current study suggest that while means tested free school meals programmes and holiday activities and food programmes may attenuate food insecurity for low-income households (3, 8, 12, 13, 38, 47, 48), many low-income families do not have access to these programmes (49–52). Parents and carers in the current study demonstrated that feeding their children healthy, nutritious food was their priority and they developed complex strategies to ensure a key children were fed. Many parents and carers reported that they made changes to the type of food they bought and their food shopping habits to stretch household budgets. Prior research has demonstrated that food insecure families cannot afford to meet recommended dietary intakes for good health (53) and are more likely to eat foods that are high in carbohydrate, fat and total energy but lower in protein and key micronutrients, which places food insecure women and children at higher risk of being overweight or obese (54–60).The over consumption of foods of low nutritional value and chronic food insecurity are both linked to adverse health outcomes, chronic diseases and premature mortality (61–66). Moreover, the findings of the current study suggest that children are potentially being exposed to much longer periods of food insecurity and potentially sub-optimal nutrition than hitherto thought.

It may also be the case that the necessity to develop and employ strategies to ensure that their children can access food throughout the year, may contribute to the poor mental health and well-being that is often experienced by food insecure adults (64, 67, 68). The toll of constantly employing strategies to mitigate food insecurity may be an important consideration for studies that have reported that holiday activity and food programmes improve parental well-being (4, 13, 48, 69).

Although data for the current study were collected in only a small number of holidays clubs from across Scotland and England, low-income parents and carers provided a detailed insight into the strategies they use to manage the competing demands of running a household and feeding their families. While parents and carers reported how free school meals programmes and holiday programmes help to attenuate food insecurity, the findings reveal that low-income families experience varying levels of food insecurity throughout the year. However, the current study does have some limitations. The small sample size may mean that the findings of the current study may not be generalisable. Thus, future research might further explore the relationship between food insecurity, food shopping habits and participation in programmes such as free school meals and holiday provision.

To conclude, the findings of the current study have important implications in terms of theory and social policy. Firstly, parents and carers report that they experience food insecurity all year and employ a range of strategies to attenuate its effects. Secondly, the findings show that parents and carers are aware of what foods constitute a nutritious diet and they try to purchase nutritious foods when financially possible. Thirdly, the findings show that level of food insecurity experienced by low-income parents and carers in the current study aligns with FAO food insecurity continuum and the academic year. Furthermore, as the level of food insecurity experienced increases, the tactics employed become more severe and parents often sacrifice their own dietary intake to feed their children; either through skipping meals entirely or eating their children's leftovers. Moreover, participation in school meal programmes and holiday activity and school food programmes are beneficial to users and communities because they attenuate, but do not completely eliminate, household food insecurity and associated negative outcomes. Furthermore, the findings add further support to three UK Parliamentary Select Committees (70–72) that concluded that low-income families simply do not have enough household income to prevent them from experiencing food insecurity.

The findings of the current study also have important implications for how governments capture data on food insecurity. For the first time, in 2021, the UK government published data on household food insecurity, collected as part of the annual Family Resources Survey (FRS). The findings of the FRS show that prior to the Covid-19 pandemic, 43% of people on Universal Credit (welfare) were food insecure, vs. 8% of the overall population. However, the FRS captures data on household food insecurity across 30 days immediately before interview (73). Our research reflects the findings of prior research that suggests that food insecurity is cyclical in nature (28–32), but for low-income families, this cycle aligns with the school calendar year. Therefore, we suggest that finer grained analyses of food insecurity data captured in representative population studies is required to capture levels of food insecurity.

Whilst we appreciate the complexity of national and local systems, policies, and interventions, we conclude by proposing that collecting and reporting the lived experiences of low-income parents and children is a crucial component to generating effective co-designed systems and social policy solutions to tackle food insecurity.

## Data Availability Statement

The raw data supporting the conclusions of this article will be made available by the authors, without undue reservation.

## Ethics Statement

The studies involving human participants were reviewed and approved by Faculty of Health & Life Sciences Ethics Committee, Northumbria University. The patients/participants provided their written informed consent to participate in this study.

## Author Contributions

JS was involved in the design, data collection and analysis of data, and writing this research. MD was involved in the design, analysis, and writing of this research.

## Conflict of Interest

The authors declare that the research was conducted in the absence of any commercial or financial relationships that could be construed as a potential conflict of interest.

## References

[1] Butcher D. The Cost of the School Holidays. Meeting the needs of low income families during school holidays (2015).

[2] Cottell J Descateaux S Coleman L. Childcare Survey 2019. (2019). Available online at: www.familyandchildcaretrust.org

[3] Stewart H Watson N Campbell M. The cost of school holidays for children from low income families. Childhood. (2018) 24:1–14. 10.1177/0907568218779130 30473595 PMC6210572

[4] Stretesky PB Defeyter MA Long MA Ritchie LA Gill DA. Holiday hunger and parental stress: evidence from North East England. Sustainability. (2020) 12:1–18. 10.3390/su12104141

[5] Forsey A. Hungry Holidays. (2017). Available online at: https://feedingbritain.files.wordpress.com/2015/02/hungry-holidays.pdf

[6] Gill O Sharma N. Food Poverty in the School Holidays. (2004). 10.12968/prps.2004.1.46.40003

[7] Gooseman A Defeyter MA Graham PL. Hunger in the primary school setting: evidence, impacts and solutions according to school staff in the North East of England, UK. Education. (2019) 48:191–203. 10.1080/03004279.2019.1602155

[8] Graham PL Crilley E Stretesky PB Long MA Palmer KJ Steinbock E. School holiday food provision in the UK: a qualitative investigation of needs, benefits, and potential for development. Front Public Health. (2016) 4:172. 10.3389/fpubh.2016.00172 27597938 PMC4992941

[9] Shinwell J. An Investigation of Holiday Club Provision: Impact on Children's Educational Attainmnet, Nutrtional Intake and Wider Familiy Benefits Nothumbria University. (2019). Available on line at: https://ethos.bl.uk/OrderDetails.do?uin=uk.bl.ethos.799718

[10] Collins AM Klerman JA Briefel R Rowe G Gordon AR Logan CW. A summer nutrition benefit pilot program and low-income children's food security. Pediatrics. (2018) 141:e20171657. 10.1542/peds.2017-1657 29592869

[11] Graham PL Stretesky PB Long MA Mann E Defeyter MA. Holiday hunger: feeding children during the school holidays. In Harman V Cappellini B Fairclough C editors. Feeding Childen Inside and Outside the Home. Oxfordshire: Routledge (2018).

[12] Holley CE Mason C. A systematic review of the evaluation of interventions to tackle children's food insecurity. Curr Nutr Rep. (2019) 8:11–27. 10.1007/s13668-019-0258-1 30762204 PMC6426823

[13] Long MA Stretesky PB Graham PL Palmer KJ Steinbock E Defeyter MA. The impact of holiday clubs on household food insecurity - a pilot study. Health Soc Care Commun. (2017) 2:1–9. 10.1111/hsc.12507 29024211

[14] Mann E Defeyter MA Widdison C. Implementing holiday provision programmes: a qualitative investigation of the experiences of senior stakeholders. Open J Soc Sci. (2020) 8:286–302. 10.4236/jss.2020.87023

[15] Miller DP. Accessibility of summer meals and the food insecurity of low-income households with children. Public Health Nutr. (2016) 19:2079–89. 10.1017/S1368980016000033 26878904 PMC10270932

[16] Molina-Azorin JF. Mixed methods research: an opportunity to improve our studies and our research skills. Eur J Manag Bus Econ. (2016) 25:37–8. 10.1016/j.redeen.2016.05.001

[17] Morgan K Melendez-Torres G Bond A Hawkins J Hewitt G Murphy S. Socio-economic inequalities in adolescent summer holiday experiences, and mental wellbeing on return to school: analysis of the school health research network/health behaviour in school-aged children survey in Wales. Int J Environ Res Public Health. (2019) 16:1107. 10.3390/ijerph16071107 30925676 PMC6480971

[18] Nord M Romig K. Hunger in the summer. J Child Poverty. (2006) 12:141–58. 10.1080/10796120600879582

[19] The Trussell Trust. Half of Children Helped by Foodbanks Over Summer Holiday Months Are Primary School Students. (2017). Available online at: https://www.trusselltrust.org/2017/07/25/half-children-helped-foodbanks-summer-holiday-months-primary-school-students/

[20] Long MA Defeyter MA Stretesky PB. Holiday Hunger in the UK: Local Responses to Childhood Food Insecurity. Routledge (2021). Available online at: https://researchportal.northumbria.ac.uk/ws/portalfiles/portal/20966249/mann.emily_phd.pdf

[21] Defeyter MA Graham PL Prince K. A qualitative evaluation of holiday breakfast clubs in the UK: views of adult attendees, children, and staff. Front Public Health. (2015) 3:156. 10.3389/fpubh.2015.00199 26322303 PMC4534775

[22] Mann E. Holiday Provision: A Mixed Methods Investigation of Holiday Clubs in Terms of Location, Implementation, Delivery and Impact. Northumbria University (2019). Available online at: http://www.frankfield.co.uk/upload/docs/Holiday%20Club%20Survey%202017.pdf

[23] Mann E Long MA Stretesky PB Defeyter MA. A question of justice: Are holiday clubs serving the most deprived communities in England? Local Environ. (2018) 23:1008–22. 10.1080/13549839.2018.1518415

[24] Mann E Defeyter MA. Holiday Club Survey 2017. Preliminary Findings. London: Northumbria University (2017). p. 1–8

[25] Braun V Clarke V. Successful Qualitative Research. London: Sage Publications Ltd. (2013).

[26] Strauss A Corbin J. Basics of Qualitative Research, 2nd ed. Rome: Sage Publications Ltd (1998).

[27] FAO. The State of Food Insecurity in the World. Colchester: FAO (2015).

[28] Burns C Cook K Mavoa H. Role of expendable income and price in food choice by low income families. Appetite. (2013) 71:209–17. 10.1016/j.appet.2013.08.018 24008182

[29] Dinour LM Bergen D Yeh MC. The food insecurity-obesity paradox: a review of the literature and the role food stamps may play. J Am Diet Assoc. (2007) 107:1952–61. 10.1016/j.jada.2007.08.006 17964316

[30] Nolsoe E. How Many Parents Struggle to Feed Their Children? (2021). Available online at: https://yougov.co.uk/topics/politics/articles-reports/2021/01/18/quarter-parents-say-there-have-been-times-where-th?utm_medium=newsletter&utm_source=internal&utm_campaign=nl-2021-02-UK-Industry-Insights&mkt_tok=eyJpIjoiT1RabFpEUTBOakV4TVRBeSIsInQiOiJQTjk2

[31] Nord M Andrews M Winicki J. Frequency and duration of food insecurity and hunger in US households. J Nutr Educ Behav. (2002) 34:194–200. 10.1016/S1499-4046(06)60093-6 12217262

[32] Tarasuk V McIntyre L Li J. Low-income women's dietary intakes are sensitive to the depletion of household resources in one month. J Nutr. (2007) 137:1980–7. 10.1093/jn/137.8.1980 17634274

[33] Loopstra R. Vulnerability to food insecurity since the COVID-19 lockdown Preliminary report. (2020). Available online at: https://foodfoundation.org.uk/wp-content/uploads/2020/04/Report_COVID19FoodInsecurity-final.pdf

[34] Adamson AJ Spence S Reed L Conway R Palmer A Stewart E. School food standards in the UK: implementation and evaluation. Public Health Nutr. (2013) 16:968–81. 10.1017/S1368980013000621 23578662 PMC10271333

[35] Au LE Rosen NJ Fenton K Hecht K Ritchie LD. Eating school lunch is associated with higher diet quality among elementary school students. J Acad Nutr Diet. (2016) 116:1817–24. 10.1016/j.jand.2016.04.010 27216647

[36] Cohen JFW Hecht AA Mcloughlin GM Turner L Schwartz MB. Universal School Meals and associations with student participation, attendance, academic performance, diet quality, food security, and body mass index: a systematic review. Nutrients. (2021) 13:911. 10.3390/nu13030911 33799780 PMC8000006

[37] Defeyter MA Graham PL Walton J Apicella T. Breakfast clubs: Availability for British schoolchildren and the nutritional, social and academic benefits. Nutr Bull. (2010) 35:245–53. 10.1111/j.1467-3010.2010.01843.x

[38] Holford A Rabe B. Impact of the Universal Infant Free School Meal Policy. Institute for Social & Economic Research (2020). p. 1–36.

[39] Jaime PC Lock K. Do school based food and nutrition policies improve diet and reduce obesity? Prev Med. (2009) 48:45–53. 10.1016/j.ypmed.2008.10.018 19026676

[40] Spence S Matthews JNS McSweeney L Rowland MK Orango P Adamson AJ. Implementation of Universal Infant Free School Meals: a pilot study in NE England exploring the impact on key stage 1 pupil's dietary intake. Public Health Nutr. (2020) 1–9. 10.1017/S1368980020004875 33261703 PMC9884742

[41] Summerbell C Crow R Wattis L. School breakfast clubs, social background and nutritional status. Topic. (2009) 44:1–4. Available online at: https://dro.dur.ac.uk/5639/

[42] NHS Scotland. Universal Infanct Free School Meals, Scotland: An Evaluation. (2016). p. 2. Available online at: http://www.healthscotland.scot/publications/universal-free-school-meals-second-monitoring-report

[43] Department for Education. Holiday Activities and Food Programme 2021. (2021). Available online at: https://www.gov.uk/government/publications/holiday-activities-and-food-programme/holiday-activities-and-food-programme-2021

[44] The Scottish Government. Holiday Meals for Children. (2019). Available online at: https://www.gov.scot/news/holiday-meals-for-children/

[45] Welsh Government. New Pilot to Tackle Food Poverty During the School Holidays. (2019). Available online at: https://gov.wales/new-pilot-tackle-food-poverty-during-school-holidays

[46] Defeyter MA Stretesky PB Sattar Z Crilley E. Evaluation of ‘ A Day Out, Not a Handout ' Holiday Provision Programme. (2018). Available online at: https://children-ne.org.uk/wp-content/uploads/2020/10/A_Day_Out_Not_a_Handout_Final_Report_Northumbria.pdf

[47] Campbell M Watson N Watters N. The Cost of School Holidays: Literature Review. (2015). p. 1–26. Available online at: https://eprints.gla.ac.uk/122643/1/122643.pdf

[48] Long MA Stretesky PB Defeyter MA Crilley E Sattar Z. Examaning the relationship between child holiday club attendance and parental wellbeing. ENUF Conf. (2020) 2020:20. 10.1016/j.puhip.2021.100122 PMC9461366 36101602

[49] Child Poverty Action Group. Two in Five UK Children Under the Poverty Line Are Not Eligible for Free School Meals. (2020). Available online at: https://cpag.org.uk/news-blogs/news-listings/two-five-uk-children-under-poverty-line-are-not-eligible-free-school-meals

[50] Dimbleby H. National food strategy: part one. Natl Food Strat. (2020) 110. Available online at: https://www.nationalfoodstrategy.org/wp-content/uploads/2020/07/NFS-Part-One-SPCP.pdf

[51] Sustain. Achieving everyone's Right to Food. (2021). Available online at: https://www.sustainweb.org/righttofood/#

[52] The Food Foundation. Children's Future Food Inquiry. (2019). Available online at: https://foodfoundation.org.uk/wp-content/uploads/2019/04/Childrens-Future-Food-Inquiry-report.pdf

[53] Taylor A Loopstra R. Too Poor to Eat: Food Insecurity in the UK. Foodfoundation.Org.Uk (2016) Available online at: www.foodfoundation.org.uk

[54] Casey PH Simpson PM Gossett JM Bogle ML Champagne CM Connell C. The association of child and household food insecurity with childhood overweight status. Pediatrics. (2006) 118:e1406–13. 10.1542/peds.2006-0097 17079542

[55] Champagne CM Casey PH Connell CL Stuff JE Gossett JM Harsha DW. Poverty and food intake in Rural America: diet quality is lower in food insecure adults in the mississippi delta. J Am Diet Assoc. (2007) 107:1886–94. 10.1016/j.jada.2007.08.003 17964307

[56] Hanson KL Connor LM. Food insecurity and dietary quality in US adults and children: a systematic review. Am J Clin Nutr. (2014) 100:684–92. 10.3945/ajcn.114.084525 24944059

[57] Johnson CM Sharkey JR Lackey MJ Adair LS Aiello AE Bowen SK. Relationship of food insecurity to women's dietary outcomes: a systematic review. Nutr Rev. (2018) 76:910–28. 10.1093/nutrit/nuy042 30184168 PMC6240001

[58] Kirkpatrick SI Tarasuk V. Food insecurity is associated with nutrient inadequacies among Canadian adults and adolescents. J Nutr. (2008) 138:604–12. 10.1093/jn/138.3.604 18287374

[59] Larson NI Story MT. Food insecurity and weight status among children US. and families: a review of the literature. Am J Prev Med. (2011) 40:166–73. 10.1016/j.amepre.2010.10.028 21238865

[60] Sharkey JR Nalty C Johnson CM Dean WR. Children's very low food security is associated with increased dietary intakes in energy, fat, and added sugar among Mexican-origin children (6-11 y) in Texas border Colonias. BMC Pediatrics. (2012) 12:16. 10.1186/1471-2431-12-16 22348599 PMC3298490

[61] Laraia BA. Food insecurity and chronic disease. Adv Nutr. (2013) 4:203–12. 10.3945/an.112.003277 23493536 PMC3649100

[62] Lawrence MA Baker PI. Ultra processed foods and adverse health outcomes. Fresh evidence links popular processed foods with a range of health risks. BMJ Clin Res. (2019) 365:l2289. 10.1136/bmj.l2289 31142449

[63] Martin MA Lippert AM. Feeding her children, but risking her health: The intersection of gender, household food insecurity and obesity. Soc Sci Med. (2012) 74:1754–64. 10.1016/j.socscimed.2011.11.013 22245381 PMC3338899

[64] Martin MSS Maddocks E Chen Y Gilman SEE Colman I. *Food insecurity and mental* illness: disproportionate impacts in the context of perceived stress and social isolation. Public Health. (2015) 132:1–6. 10.1016/j.puhe.2015.11.014 26795678

[65] Rico-campà A Martínez-gonzález MA Alvarez-alvarez I Mendonça RD Fuente-arrillaga C Gómez-Donoso C. Association between consumption of ultra-processed foods and all cause mortality : SUN prospective cohort study. BMJ. (2016) 365:1949. 10.1136/bmj.l1949 PMC6538973 31142450

[66] Vozoris NT Tarasuk VS. Household food insufficiency is associated with poorer health. J Nutr. (2003) 133:120–6. 10.1093/jn/133.1.120 12514278

[67] Heflin CM Siefert K Williams DR. Food insufficiency and women's mental health: Findings from a 3-year panel of welfare recipients. Soc Sci Med. (2005) 61:1971–82. 10.1016/j.socscimed.2005.04.014 15927331

[68] Melchior M Chastang JF Falissard B Galéra C Tremblay RE Côté SM. Food insecurity and children's mental health: a prospective birth cohort study. PLoS ONE. (2012) 7:e52616. 10.1371/journal.pone.0052615 23300723 PMC3530436

[69] Stretesky PB Defeyter MA Long MA Sattar ZZ Crilley E. Holiday clubs as community organisations. ANALS Am Acad Polit Soc Sci. (2020) 689:129–48. 10.1177/0002716220917657

[70] House of Commons. Welfare Safety Net. London: House of Commons (2019). Available on line at: http://www.publications.parliament.uk/pa/cm201011/cmselect/cmworpen/472/472vw60.htm

[71] House of Commons. Work and Pensions and Education Committees - Oral Evidence: School Holiday Poverty. London: House of Commons (2019). Available online at: http://data.parliament.uk/writtenevidence/committeeevidence.svc/evidencedocument/work-and-pensions-committee/school-holiday-poverty/oral/103517.pdf

[72] House of Lords. Hungry for Change: Fixing the Failures in Food. London (2020). Available online at: https://committees.parliament.uk/publications/1762/documents/17092/default/

[73] Department for Work and Pensions. Family Resources Survey: Background Information and Methodology. (2021). Avaiable online at: https://www.gov.uk/government/statistics/family-resources-survey-financial-year-2019-to-2020/family-resources-survey-background-information-and-methodology

